# Evaluating alternative compounds for strongyloidiasis therapy: Novel insights from larval migration inhibition test

**DOI:** 10.1371/journal.pntd.0012532

**Published:** 2024-10-07

**Authors:** María Cambra-Pellejà, Elora Valderas-García, Rafael Balaña-Fouce, Jennifer de la Vega, Esther del Olmo, Jennifer Antwi-Ekwuruke, Lara Linnemann, Lennart Heepmann, Minka Breloer, María Martínez-Valladares

**Affiliations:** 1 Departamento de Sanidad Animal, Facultad de Veterinaria, Universidad de León, Campus de Vegazana, León, Spain; 2 Instituto de Ganadería de Montaña (CSIC-Universidad de León), Grulleros, León, Spain; 3 Departamento de Ciencias Biomédicas, Institute of Biomedicine (IBIOMED), Universidad de León, León, Spain; 4 Departamento de Ciencias Farmacéuticas: Química Farmacéutica, Facultad de Farmacia, Universidad de Salamanca, CIETUS, IBSAL, Salamanca, Spain; 5 Bernhard Nocht Institute for Tropical Medicine, Section Interface, Helminth Immunology Group Hamburg, Germany; 6 Department of Biology, University of Hamburg, Hambug, Germany; Centre for Tropical Diseases, ITALY

## Abstract

Strongyloidiasis is a neglected tropical disease estimated to affect more than 600 million people worldwide. Recently, the World Health Organization road map on neglected tropical diseases 2021–2030 has put the focus on strongyloidiasis, including this disease within its mass drug administration campaigns. With the use of ivermectin in extensive treatment of all populations at-risk, identifying effective therapeutic alternatives is crucial in case ivermectin resistance arises. The objective of the present study was the development of a larval migration inhibition assay to evaluate the anthelmintic efficacy of commercial drugs and diamine and aminoalcohol derivatives against infective *Strongyloides ratti* third stage larvae. Through this technique, we successfully screened and estimated the *in vitro* anthelmintic efficacy of six commercial drugs, seven diamine derivatives and eight aminoalcohol derivatives. Unexpectedly, the half-maximal effective concentration of ivermectin and moxidectin (2.21 and 2.34 μM, respectively) were observed as the highest value obtained among all commercial drugs tested by this *in vitro* technique. Moreover, some diamine and aminoalcohol derivatives showed superior efficacy inhibiting *S*. *ratti* motility compared to ivermectin, with five compounds (AA23, AA34, AO2 AO7 and AO14b) also displaying selectivity indexes on HepG2 and Caco2 higher than 1. These findings underscore the potential of these derivatives as promising alternatives for strongyloidiasis treatment, warranting further investigation and *in vivo* efficacy assessment.

## Author summary

Strongyloidiasis, a parasitic infection affecting over 600 million people worldwide, is increasingly recognized in global health initiatives. Caused by the nematode *Strongyloides stercoralis*, it predominantly occurs in tropical and subtropical regions. Transmission results through contact with soil contaminated by infectious larvae, leading to a spectrum of clinical symptoms from mild gastrointestinal discomfort to potentially life-threatening complications, especially in individuals with weakened immune systems. Despite its designation as a neglected tropical disease, concerns regarding the development of drug resistance highlight the need to explore alternative treatment strategies. In our study, we employed innovative methods to evaluate the *in vitro* efficacy of various drug candidates against *Strongyloides ratti* larvae, representing a crucial step in identifying novel therapies for human strongyloidiasis. Our findings revealed that some of the novel compounds tested, showed superior efficacy in inhibiting parasite motility, suggesting potential benefits for patient treatment outcomes. Our study underscores the urgent need for diversified treatment approaches in combating strongyloidiasis.

## Introduction

Strongyloidiasis is a Neglected Tropical Disease (NTD) estimated to affect more than 600 million people worldwide, even though evidence indicates that these numbers may be underestimated [1]. The South-East Asian, African, and Western Pacific Regions accounted for the majority of the global infections [1]. The unique autoinfective cycle of *Strongyloides stercoralis* favours the perpetuation of the infection, even lasting decades in humans [2]. *S*. *stercoralis* usually causes mild, asymptomatic, and chronic infections, where individuals may be unaware of being infected [2]; however, when symptoms do manifest, dermatologic, intestinal, and respiratory manifestations are most common [2]. In certain cases, such as in immunosuppressed individuals, strongyloidiasis can be life threatening if not promptly and correctly treated [2]. This includes the risk of hyperinfection syndrome, characterized by a significant increase in parasite burden, which can lead to systemic dissemination and carries 87% fatality rate [3]; hence, the importance of optimal treatment cannot be overstated. In fact, the rodent-specific *S*. *ratti* and *S*. *venezuelensis* can be used to study larval migration and immune response *in vivo* in the mouse system [4,5].

Recently, the World Health Organization (WHO) road map on NTDs 2021–2030 has set the new targets and milestones to achieve NDTs control and eradication over the next decade [4]. In this road map, and for the first time, the WHO put the focus on strongyloidiasis, including this disease within its mass drug administration (MDA) campaigns [6]. Ivermectin (IVM) is the drug of choice for strongyloidiasis treatment, and it was included in the WHO Model List of Essential Medicines for this purpose [7]. IVM is considered to be highly effective for treating the infection [6]; however, albendazole and thiabendazole are also used, especially when IVM is difficult to procure, but they show reduced effectiveness [6,8]. With the use of IVM in MDA campaigns against strongyloidiasis, we think that a better knowledge about its efficacy in these programs will be acquired. However, considering the limited number of drugs available for treating strongyloidiasis, the extensive treatment of all populations at risk, and the zoonotic nature of the disease [9,10]–where animals act as reservoirs and are treated with the same drugs used for humans–identifying effective therapeutic alternatives becomes crucial in the event of IVM resistance.

Diamine (AA) and aminoalcohol (AO) derivatives are compounds structurally considered as simplified sphingosine pseudo-analogues [11]. Sphingosine is a ubiquitous constituent of eukaryotic membranes involved in numerous cellular processes such as growth, movement, programmed cell death, self-degradation, autophagy, ageing and immune system reactions, and its analogues or derivatives can interfere with these processes leading to parasite death [12]. Therefore, some AA and AO derivatives have been already tested against other different species of helminths by *in vitro* techniques with the aim to evaluate their potential anthelmintic efficacy [13–15].

*In vitro* tests are well-established procedures in the veterinary field to evaluate the efficacy of the treatment against nematodes infecting livestock, as these tools are an easy and cost-effective approach for drug testing [11,16,17]. Different types of *in vitro* assays have been developed depending on which parasitic stage is the most desired to be tested (e.g., eggs, larvae, adult worms). Since 2001, some *in vitro* tests have been used with different stages of both, the laboratory models *S*. *ratti* and *S*. *venezuelensis*, but also with the infective third stage larvae (L3) of the human pathogenic *S*. *stercoralis* [18–26] (Table 1). Most of these studies have used the Larval Motility Test (LMT) as the main evaluating tool, which assesses the *in vitro* motility of the larvae after their incubation with the tested compounds.

**Table 1:** Main characteristics of Larvae Motility Test (LMT) and Larval Migration Inhibition Test (LMIT) performed with different *Strongyloides* species alongside half-maximal effective concentration (EC_50_) values for ivermectin (IVM) after 24 h of incubation.

*In vitro* test	Species	Incubation T^a^	IVM EC_50_ (24h)	Reference
**LMT**	*S*. *ratti*	No data	0.060 μM	18
**LMT**	*S*. *ratti*	37°C	2.400 μM	19
**LMT**	*S*. *venezuelensis*	37°C	2.600 μM	19
**LMT**	*S*. *ratti*	20°C	1.200 μM	20
**LMT**	*S*. *stercoralis*	37°C	9.760 μM	21
**LMT**	*S*. *venezuelensis*	28°C	11.500 μg/ml	22
**LMT**	*S*. *ratti*	Room T^a^	0.080 μM	23
**LMT**	*S*. *stercoralis*	No data	2.250 mM	24
**LMT**	*S*. *ratti*	37°C	2.200 μM	25
**LMT**	*S*. *venezuelensis*	37°C	2.300 μM	19.25
**LMIT** *	*S*. *ratti*	20–23°C	No data	26

* It was not considered as tool for drug testing in that paper and it is a slightly different technique than what is reported in the present paper.

Under this context, the objective of the present study was to evaluate the anthelmintic efficacy of commercial drugs and newly synthesized compounds (AA and AO derivatives) against *S*. *ratti* L3 using a new *in vitro* test, the Larval Migration Inhibition Test (LMIT), and the LMT. The LMIT is based on the count of active larvae that are able to migrate through a mesh after their incubation with the compound of interest. *In vitro* efficacy results using both techniques were compared and discussed.

## Methods

### Ethics statement

Wistar rats were kept in the animal facilities of the Bernhard Nocht Institute for Tropical Medicine (BNITM) and experimental protocols were approved by Federal Health Authorities of the State of Hamburg (permission number A20/2020).

### *S*. *ratti* life cycle and L3 production

The *S*. *ratti* cycle was maintained in Wistar rats and L3 were purified from charcoal faeces cultures of infected rats as described [23]. Purified L3 were washed three times in phosphate buffered saline supplemented with penicillin and streptomycin (100U/ml) (PBS/PenStrep) (Thermo Fisher Scientific GmbH, Germany). To this end 10 mL PBS Pen/Strep was added to the L3 in 15 mL Falcontubes. L3 were allowed to sediment for 20 min, supernatant was removed and 10 mL PBS/PenStrep was added for the next washing step. A final washing step was performed using 10 ml PBS/PenStrep supplemeted with Gentamicin (1mg/mL) (Capricorn Scientific GmbH, Germany) under a laminar flow hood. To avoid fungal contamination the larvae were then incubated in PBS/PenStrep supplemented with Amphotericin B (2 μg/mL) (Gibco, Thermo Fisher Scientific GmbH, Germany) for another 20 min, washed and resuspended in dH_2_O for further LMT or LMIT.

### Commercial drugs and chemical compounds

**Fig 1:**
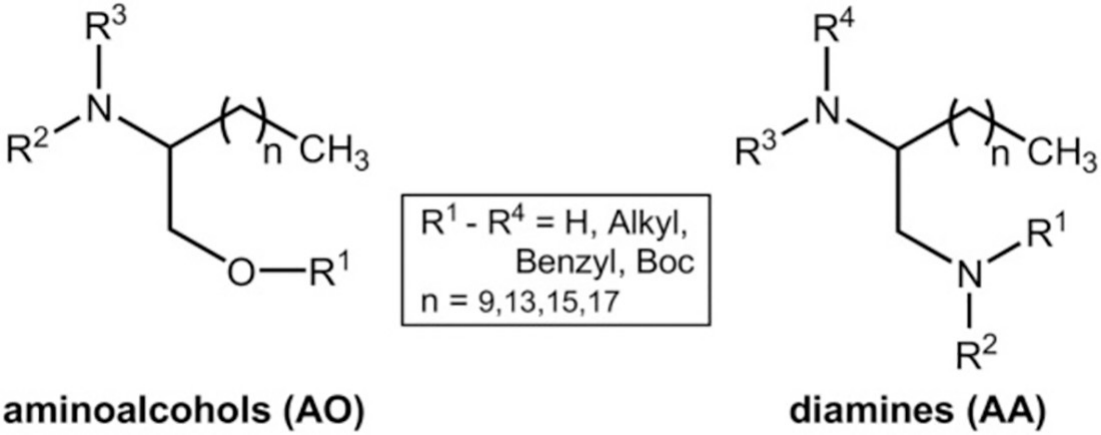
General structures for the tested aminoalcohol (AO) and diamine (AA) derivatives [11].

The commercial anthelmintic drugs IVM, moxidectin (MOX), abamectin (ABA), doramectin (DOR), milbemycin (MIL), levamisole (LEV), and pyrantel pamoate (PYR) (Sigma-Aldrich, Spain) were tested; also, a bunch of benzimidazoles such as albendazole, fenbendazole, mebendazole and oxfendazole (Sigma-Aldrich, Spain). Seven AA and nine AO derivatives structurally related to sphingosine were synthesized as in previous studies [27,28]. All AA and AO derivatives were named consistently with a previous study conducted by our research group [11] (Fig 1), except a compound AO14, which was designated in accordance with the nomenclature used in a separate study, and in this study is named AO14b [29]. Chemical compound structures are shown in Fig 1.

Stock solutions of commercial drugs and AA and AO derivatives were prepared in dimethyl sulfoxide (DMSO, ≥99.9%, Merk, Spain), while the final dilutions were made in order to maintain a maximum concentration of 0.5% (v/v) DMSO in each well.

### Larval motility test

The LMT was performed to evaluate only the efficacy of the commercial drugs similarly to the methods described by [11,30]. Per well, 20 L3 were included in a final volume of 200 μL of distilled water (dH_2_O) in a 24-well plate. In each well, the drug or compound was added to get the final concentrations showed in Table 2. Each plate also included three positive control wells, with dead larvae, killed by incubation at 75°C for 30 min, and three negative control wells, with DMSO 0.5% v/v. After 24 h of incubation in the dark at 37°C, motility was scored after the addition of 100 μL dH_2_O at 50°C [19]. A flowing scoring system ranging from 0 to 4 was applied [30]. In this score, 4: fast and continuous movement, 3: slower but continuous movements, 2: slow and discontinuous movements, 1: sporadic movements at posterior/anterior body parts only, 0: no movement was recorded (see Supplementary information S1 Videos for representative videos). The scoring was performed in a blinded manner. The activity was expressed as the percentage of larvicidal activity using the following formula: percentage of larvicidal activity = (percentage of viability inhibition per well / percentage of viability inhibition in control well) × 100 [9]. The half-maximal effective concentration (EC_50_) values were determined by plotting the percentage of larvicidal activity versus the concentration of each compound. Concentrations were evaluated in triplicate and at least in two different days.

**Table 2:** Serial concentrations of commercial drugs tested in the Larval Motility Test.

Commercial drug	Higher concentration (μM)	Lowest concentration (μM)	Dilution factor
**IVM and MOX**	8	0.125	1:2
**ABA**	6.250	0.098	1:2
**DOR**	50	0.781	1:2
**LEV**	1	0.016	1:2
**PYR**	2	0.031	1:2

### Larval migration inhibition test

The final protocol was conducted based on previous studies [11,16]. Per well, a total of 400 L3 were included in a final volume of 1.4 mL of dH_2_O in a 24-well plate. Each plate also included one control well with 400 dead L3 (for that, L3 were incubated at 75° for 30 min), and a negative control well, with 400 L3 and DMSO 0.5% v/v. Initially, all commercial drugs were screened at a concentration of 50 μM, and the newly synthetized compounds at 50 μM and 10 μM. After 24 h of incubation of the L3 with the drug or compound in the dark at 37°C, L3 from each well were divided into 3 volumes of 400 μL each (containing between 100–150 L3) which were transferred into three different wells (three replicates) of a 96-wells MultiScreen-Mesh Filter Plate (Merk, Spain). The plate was left for 24 h at 37°C to allow the motile L3 to migrate through 20 μm mesh size for counting. Then, the percentage of larval migration was determined. The activity of each drug or AA/AO derivative was expressed as the percentage of larval migration inhibition using the following formula: percentage of larval migration inhibition = 100 –(percentage of larval migration for each drug/compound) [9]. EC_50_ was calculated for those commercial drugs and compounds with an activity higher than 65% at 50μM and 10μM, respectively. In each well, the drug or AA/AO derivative was added to get the final concentrations showed in Table 3. EC_50_ values were determined by plotting the percentage of larval migration inhibition versus the concentration of each compound. Each concentration was evaluated in triplicate and at least in two different days.

**Table 3:** Serial concentrations of commercial drugs and compounds tested in the Larval Migration Inhibition Test.

Commercial drug / Compound	Higher concentration (μM)	Lowest concentration (μM)	Dilution factor
**IVM and ABA**	100	0.024	1:4
**MOX and DOR**	50	0.012	1:4
**LEV**	2	0.003	1:3
**PYR**	12.500	0.195	1:2
**AA25 and AO7**	3	0.047	1:2
**AA27 and AO14**	40	0.625	1:2
**AO8**	5	0.439	1:1.5
**AO9**	10	1	1μM*
**All other AA and AO derivatives**	10	0.156	1:2

* less as to the previous concentration.

### Selectivity Indexes of chemical compounds

The selectivity indexes (SIs), an estimation of safety, were calculated for each tested compound along with two commercial drugs, IVM and LEV. To achieve this, the half-maximal effective cytotoxic concentration (CC_50_) of each compound was divided by its respective EC_50_ value as determined in this study. The higher SI value, the safer the compound is considered to be [31]. Cytotoxicity of all compounds was previously assessed in a different study on human Caco-2 and HepG2 cell cultures [9].

## Results

### *In vitro* results of commercial drugs using the LMIT and LMT

The efficacy of the commercially available anthelmintics IVM, MOX, ABA, DOR, MIL, LEV and PYR was tested using both LMIT and the LMT. The initial screening showed that all drugs except MIL and all benzimidazoles, had a larval migration inhibition activity higher than 60% at a concentration of 50 μM (Table 4). EC_50_ values ranged from 0.51 μM to 2.34 μM, with MOX having the highest EC_50_ (Table 4). In direct comparison, the EC_50_ values using the LMT resulted in general lower than by the LMIT, ranging from 0.23 μM to 1.62 μM, with DOR being the least effective drug (Table 4). Dose-response curves for each of the compounds are shown in the Supplementary Material (Figs A and B in S1 Text).

**Table 4:** Efficacy results of commercial drugs tested against *S*. *ratti* L3 using both Larval Migration Inhibition Test (LMIT) and Larval Motility Test (LMT). Along with cytotoxicity data and their respective Selectivity Indexes (SIs) of LEV and IVM. Cytotoxicity data (CC_50_) came from a previous work [11].

Drug	% of efficacy at 50 μM by LMIT	EC_50_ by LMIT (μM)	EC_50_ by LMT (μM)	CC_50_ in HepG2 (μM)	CC_50_ in Caco2 (μM)	SI in HepG2 LMIT	SI in Caco2 LMIT	SI in HepG2 LMT	SI in Caco2 LMT
**IVM**	66.7 ± 07.4	02.21 ± 1.44	00.51 ± 00.53	20.86 ± 0.78	24.74 ± 0.81	09.44	11.19	40.90	48.51
**MOX**	71.2 ± 15.1	02.34 ± 1.32	00.25 ± 00.29	-	-	-	-	-	-
**ABA**	68.1 ± 07.7	01.85 ± 1.16	00.39 ± 00.31	-	-	-	-	-	-
**DOR**	61.7 ± 24.1	01.12 ± 1.38	01.62 ± 01.65	-	-	-	-	-	-
**LEV**	100 ± 00.0	00.63 ± 1.11	00.66 ± 00.65	02.43 ± 0.35	02.91 ± 0.97	03.86	04.62	03.68	04.41
**PYR**	100 ± 00.0	00.51 ± 1.09	00.23 ± 01.33	-	-	-	-	-	-

### *In vitro* results of diamine and aminoalcohol derivatives using the LMIT

The initial screening of AA derivatives with LMIT at 10 μM revealed that all tested compounds exhibited larval migration inhibition activity, with most percentages exceeding 70% (Table 5). Their corresponding EC_50_ values ranged from 0.81 μM to 8.04 μM (Table 5). Regarding AO derivative, six out of the nine compounds showed a larval migration inhibition activity higher than 90% at 10 μM, with EC_50_ values ranging from 1.14 μM to 15.34 μM (Table 5). Compounds showing higher EC50 values than IVM are AA23 (0.81 μM), AA34 (1.63μM), AO2 (1.72 μM), AO7 (1.14 μM) and AO14b (1.30 μM). Dose-response curves for each of the compounds are shown in the Supplementary Material (Fig C and D in S1 Text).

**Table 5:** Efficacy results of diamine and aminoalcohol derivatives tested against *S*. *ratti* L3 with Larval Migration Inhibition Test. along with cytotoxicity data and their respective Selectivity Indexes (SIs). Cytotoxicity data (CC_50_) came from a previous work [11,29]*. Data are presented as mean ± standard error.

Compounds	% of efficacy at 10 μM by LMIT	EC_50_ by LMIT (μM)	CC_50_ in HepG2 (μM)	CC_50_ in Caco2 (μM)	SI in HepG2	SI in Caco2
**Diamine derivatives**						
**AA23**	100 ± 00.0	00.81 ± 04.89	25.20 ± 00.33	30.24 ± 01.56	31.11	37.33
**AA24**	100 ± 00.0	02.60 ± 01.55	06.67 ± 00.32	>20	02.57	>7.69
**AA25**	100 ± 00.0	02.36 ± 01.23	07.37 ± 00.31	09.11 ± 00.66	03.12	03.86
**AA27**	82 ± 27.1	08.04 ± 01.12	05.05 ± 02.48	18.31 ± 01.31	0.63	02.28
**AA33**	84 ± 13.9	02.70 ± 01.73	15.88 ± 03.73	46.63 ± 00.98	05.88	17.27
**AA34**	100 ± 00.0	01.63 ± 01.10	09.97 ± 01.35	>15	06.12	>9.20
**Aminoalcohol derivatives**						
**AO2**	97 ± 05.5	01.72 ± 01.12	07.78 ± 00.33	12.59 ± 00.06	04.52	07.32
**AO5**	61 ± 22.9	15.34 ± 06.16	24.32 ± 00.23	54.80 ± 07.78	01.59	03.57
**AO6**	99 ± 01.1	02.44 ± 01.20	07.70 ± 01.27	>37.5	03.16	>15.37
**AO7**	100 ± 00.0	01.14 ± 01.95	09.24 ± 00.32	09.22 ± 00.54	08.11	08.09
**AO8**	97 ± 32.1	03.95 ± 01.10	09.13 ± 00.56	13.01 ± 00.27	02.31	03.29
**AO9**	82 ± 18.3	05.33 ± 02.24	08.08 ± 00.30	13.48 ± 00.42	01.52	02.53
**AO14**	71 ± 36.1	04.32 ± 01.29	06.48 ± 00.18	>20	01.50	>4.63
AO14b*	99 ± 01.3	01.30 ± 01.12	13.68 ± 01.13	>15	10.52	>11.54
**AO21**	94 ± 09.9	05.32 ± 01.23	06.69 ± 01.03	10.85 ± 02.43	01.26	02.04

### Selectivity Indexes of chemical compounds

Among all compounds tested, only one AA (AA23) and ones AO (AO14b*) reached SIs over 10 in both cell lines, while AA33 and AO6 only exceeded the SI value of 10 in Caco-2 cells. The remaining compounds showed SIs lower than 10, but above one, except for AA27, which had an SI of 0.63 in hepatocytes (Table 5). Additionally, the SIs for IVM were 40.90 and 48.51 in HepG2 and Caco-2 cells, respectively. LEV showed values lower than 10 in both cell lines (Table 4).

## Discussion

The WHO, with its road map on NTDs 2021–2030, put the focus on strongyloidiasis including the disease within its mass drug administration (MDA) campaigns [6]. Since a limited number of drugs are available to treat strongyloidiasis [6,8], in this work we set up an *in vitro* technique, LMIT, to evaluate potential therapeutic alternatives for its treatment. The activity of commercially available drugs and novel compounds such as AA and AO derivatives were tested *in vitro* using the LMIT but also the LMT, an *in vitro* test used previously in several studies with L3 from different *Strongyloides* species [18–26].

Both LMIT and LMT are *in vitro* techniques easy to be performed in laboratories from high-income resource countries. However, some attempts of *in vitro* techniques such as LMT are being applied in laboratories from low-resource settings too [32]. Initially the *in vitro* activity of commercial drugs with anthelmintic activity, used mainly in the veterinary medicine, was tested with the aim to compare the feasibility of the LMIT, in comparison with the LMT. Our results revealed that the EC_50_ values obtained when testing the same commercial drugs were lower with LMT than LMIT. This could be explained by the fact that the LMT measures all types of motilities, ranging from scores 1 to 4. In contrast, the majority of larvae actively penetrating the meshes in the LMIT are supposed to be those that showed fast and continuous movement (score 4) and slower but continuous movements (score 3). Therefore, larvae with slow and discontinuous movements (score 2) and with sporadic movements of the posterior third (score 1) may not be counted in the LMIT. LMIT offers improved precision by eliminating the subjectivity of user assessments involved in LMT motility scoring. Moreover, LMIT reduces the need for highly specialized technicians, is less time-consuming and allows the screening of a higher number of L3 in each assay. Therefore, we consider that with all of these characteristics, the LMIT is an accurate and easy-to-use technique to measure the activity of compounds against *S*. *ratti* L3.

Regarding the activity of the commercial drugs, unexpectedly, IVM EC_50_ was one of the highest value obtained (2.21 μM), along with MOX (2.34 μM) among all of them by LMIT. We compared the results obtained in the present work using LMIT with those results from Satou et al. [19], who used LMT under identical conditions for evaluating the efficacy of IVM against *S*. *ratti* L3. Our findings revealed an IVM EC_50_ of 2.21 μM, closely aligning with their EC_50_ of 2.4 μM. These results corroborate the robustness of the LMIT. The highest activities with both LMIT and LMT in commercially available drugs was obtained for PYR (0.51 μM and 0.23 μM, respectively). In line with our findings, Keiser and Häberli [33] conducted a study examining 1,600 FDA-approved drugs *in vitro* against *S*. *ratti* L3 in which both commercial drugs were included to be tested. They identified LEV as a promising anthelmintic drug, prompting them to further assess its efficacy *in vivo* [33]. Their results suggest that LEV holds promise as a potential alternative drug for strongyloidiasis and they recommended it being used in exploratory clinical trials to evaluate its efficacy in humans [33]. In our study LEV showed very good efficacies by both methods, with EC_50_ values lower than 1 μM, 0.63 μM by LMIT and 0.66 μM by LMT. As regards of PYR, even though it showed 100% efficacy on *S*. *ratti* L3 after 72 hours incubation in Keiser and Häberli [33], it only had moderate activity when being tested against *S*. *ratti* adults, and therefore they did not go further testing it *in vivo* [33].

Regarding LMIT results, 5 compounds (AA23, AA34, AO2, AO7 and AO14b) showed EC_50_ values lower than IVM (2.21 μM). It is important to mention that from a chemical point of view, the structure of IVM and AA/AO are very different. IVM is a 16-membered macrocyclic lactone with various substitutions where the presence of oxygen atoms predominates, and in the case of the AA and AO compounds, they consist of a long linear chain of 14, 16 or 18 carbons and heteroatoms of nitrogen and oxygen substituted with small/medium fatty chains or aromatics. Moreover, the size of these molecules is also very different, IVM with 875 atomic mass units (amu) and AA/AO between 220 and 396 amu.

In terms of compound safety, AA23, AA33, AO6 and AO14b displayed SI values higher than 10 in both or at least one of the cell lines (Tables 4 and 5). Additionally, A23 reached the highest SI among all compounds in HepG2 cells with a value of 31.11, while in Caco-2 cells, achieved a SI of 37.33. Interestingly, AA34, with SIs of 6.12 in HepG2 and >9.20 in Caco2 cells, resulted safe *in vivo* when administered to mice in a single oral dose of 250 mg/kg body weight [34]. Hence, compounds displaying higher SI values than AA34 in the present study are likely to be safe for *in vivo* application. However, further validation is essential.

All derivative compounds tested in the present study underwent a prior screening at 50 μM on *Teladorsagia circumcincta* larvae, a gastrointestinal nematode infecting sheep. This screening assessed 34 compounds, selecting only those showing larvicidal activities exceeding 80%. This selection process could explain the robust activity observed in all AA and AO derivatives, effectively inhibiting *S*. *ratti* L3 motility in the present study.

This type of AA and AO derivatives had been tested *in vitro* by other researchers in studies against helminth parasites, including *Schistosoma mansoni* [13] and *Echinococcus granulosus* [14]. In *in vitro* tests against *S*. *mansoni* adult worms, certain AO achieved 100% mortality at a concentration of 10 μM, while others showed a reduction in egg production in female adult parasites compared to negative controls [13]. Liu et al. [14] work on *E*. *granulosus* demonstrated the efficacy of some AO derivatives *in vitro* against protoscoleces and germinal cells, with EC_50_s ranging from 3 to 22 μM, slightly higher than the results obtained with our AO compounds. They also performed some ultrastructural analyses through scanning electron microscopy and transmission electron microscopy, revealing alterations induced by AO derivatives in *E*. *granulosus* [14].

In addition, some of the compounds used in the present study were also subjected to *in vitro* screening against other helminth parasites, including the gastrointestinal nematodes *Trichuris muris* [29] and *S*. *venezuelensis* [15]. Regarding *T*. *muris*, AO14b emerged as the most potent compound within the AO and AA series against L1, however it showed low activity against adults, 20% of activity at 10 μM. In the case of *S*. *venezuelensis* L3, Legarda Ceballos et al. [15] identified AO9, AO6, AA23, AA24 and AA33 as the most potent derivatives, with EC_50_ values ranging between 30 and 40 μM *in vitro* [15]. Despite these relatively high values, when administered at 20 mg/kg for 5 days, AO9 and AA24 were able to produce a reduction in the number of parthenogenetic female adults in the gut of mice infected with *S*. *venezuelensis* by day 7 post infection, along with a reduction in the number of eggs per gram faeces. This suggests that AO and AA derivatives induce direct ultrastructural alterations in helminths, leading to reduced viability and egg production by the adult female parasite. AO and AA are alkylphospholipid compounds that have the capability to interact with membrane lipids, facilitating their entry into the parasite and potentially causing alterations in metabolism and cellular integrity [35]. In long-chain AO also a pro-apoptotic mechanism has been described [36]. Furthermore, some of these compounds were also evaluated against protozoa parasites such as *Leishmania* spp. [37,38], wherein AA33 and AA34 were observed to effectively kill promastigotes of different species. Also, del Olmo et al [27] reported potent *in vitro* activity of AO7 against *Trypanosoma brucei*, with an EC_50_ value around 0.5 μM.

In conclusion, a screening protocol for compounds in *S*. *ratti* L3 has been established that allows a series of compounds to be evaluated in a more objective way. In this sense, we have identified AO and AA derivatives that exhibit superior *in vitro* activity compared to IVM. Therefore, we believe that pursuing further investigation and assessing the *in vivo* efficacy of these compounds should be the following steps.

## Supporting information

S1 Videos Motility scoring video. (PPTX)

S1 Text Fig A. Dose-response curves for commercial drugs derived from the Larval Migration Inhibition Test (LMIT). Y-axis is the efficacy percentage (%). The standard error is shown by the error bars. Fig B. Dose-response curves for commercial drugs derived from the Larval Mortality Test (LMT). Y-axis is the efficacy percentage (%). The standard error is shown by the error bars. Fig C. Dose-response curves for diamine (AA) derivatives derived from the Larval Migration Inhibition Test (LMIT). Y-axis is the efficacy percentage (%). The standard error is shown by the error bars. Fig D. Dose-response curves for aminoalcohol (AO) derivatives derived from the Larval Migration Inhibition Test (LMIT). Y-axis is the efficacy percentage (%). The standard error is shown by the error bars.
(DOCX)
