# Supplementary material for: Evaluating alternative compounds for strongyloidiasis therapy: Novel insights from larval migration inhibition test
Source: PLoS Negl Trop Dis. 2024 Oct 7;18(10):e0012532. doi: 10.1371/journal.pntd.0012532 (PMC11458022; doi:10.1371/journal.pntd.0012532)
Supplement: S1 Videos — (PPTX) [file pntd.0012532.s001.pptx]

## Slide 1
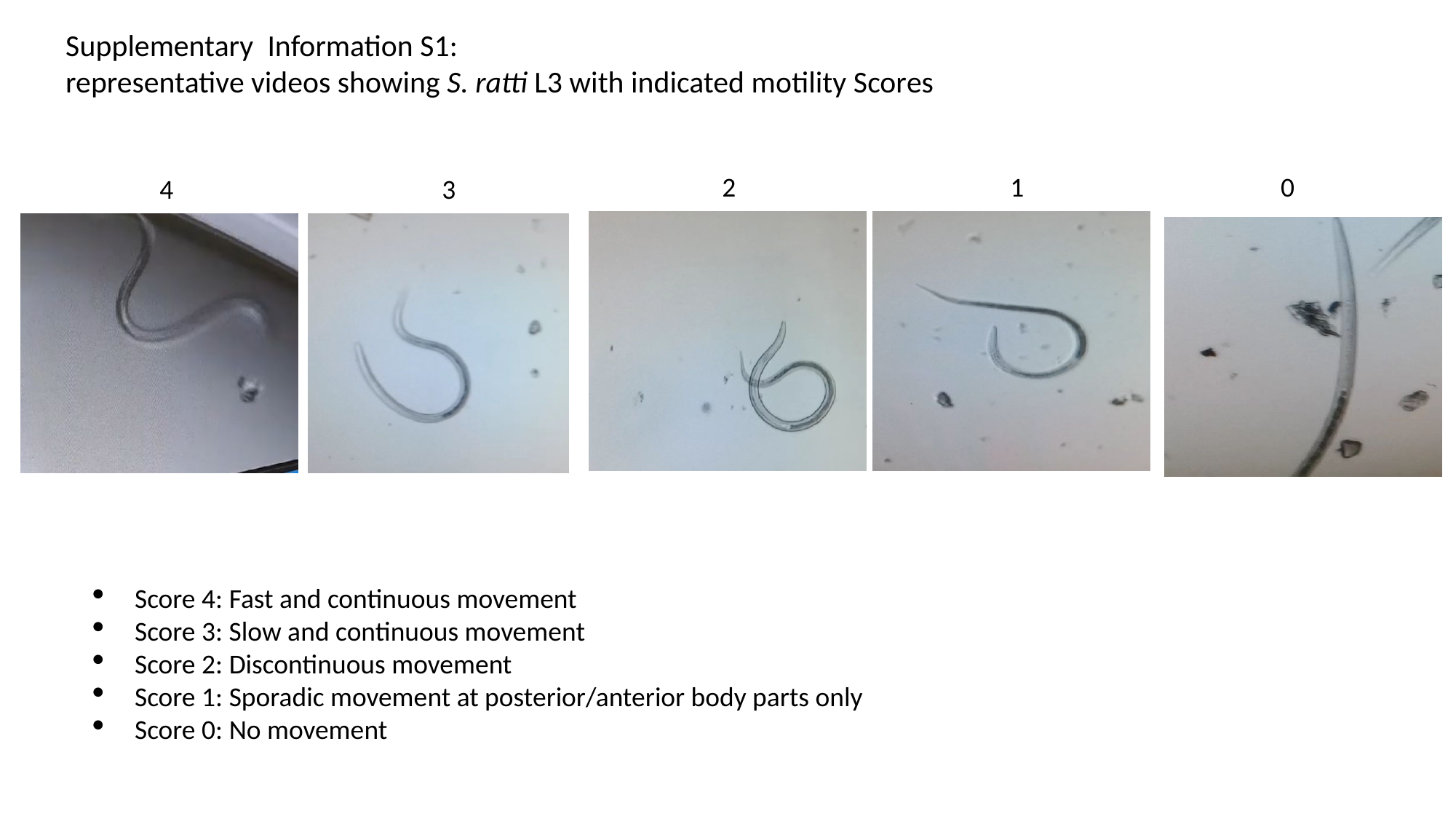

Supplementary Information S1:
representative videos showing S. ratti L3 with indicated motility Scores
2
1
0
4
3
Score 4: Fast and continuous movement
Score 3: Slow and continuous movement
Score 2: Discontinuous movement
Score 1: Sporadic movement at posterior/anterior body parts only
Score 0: No movement
1
