# Supplementary material for: Evaluating alternative compounds for strongyloidiasis therapy: Novel insights from larval migration inhibition test
Source: PLoS Negl Trop Dis. 2024 Oct 7;18(10):e0012532. doi: 10.1371/journal.pntd.0012532 (PMC11458022; doi:10.1371/journal.pntd.0012532)
Supplement: S1 Text — Fig A. Dose-response curves for commercial drugs derived from the Larval Migration Inhibition Test (LMIT). Y-axis is the efficacy percentage (%). The standard error is shown by the error bars. Fig B. Dose-response curves for commercial drugs derived from the Larval Mortality Test (LMT). Y-axis is the efficacy percentage (%). The standard error is shown by the error bars. Fig C. Dose-response curves for diamine (AA) derivatives derived from the Larval Migration Inhibition Test (LMIT). Y-axis is the efficacy percentage (%). The standard error is shown by the error bars. Fig D. Dose-response curves for aminoalcohol (AO) derivatives derived from the Larval Migration Inhibition Test (LMIT). Y-axis is the efficacy percentage (%). The standard error is shown by the error bars. (DOCX) [file pntd.0012532.s002.docx]

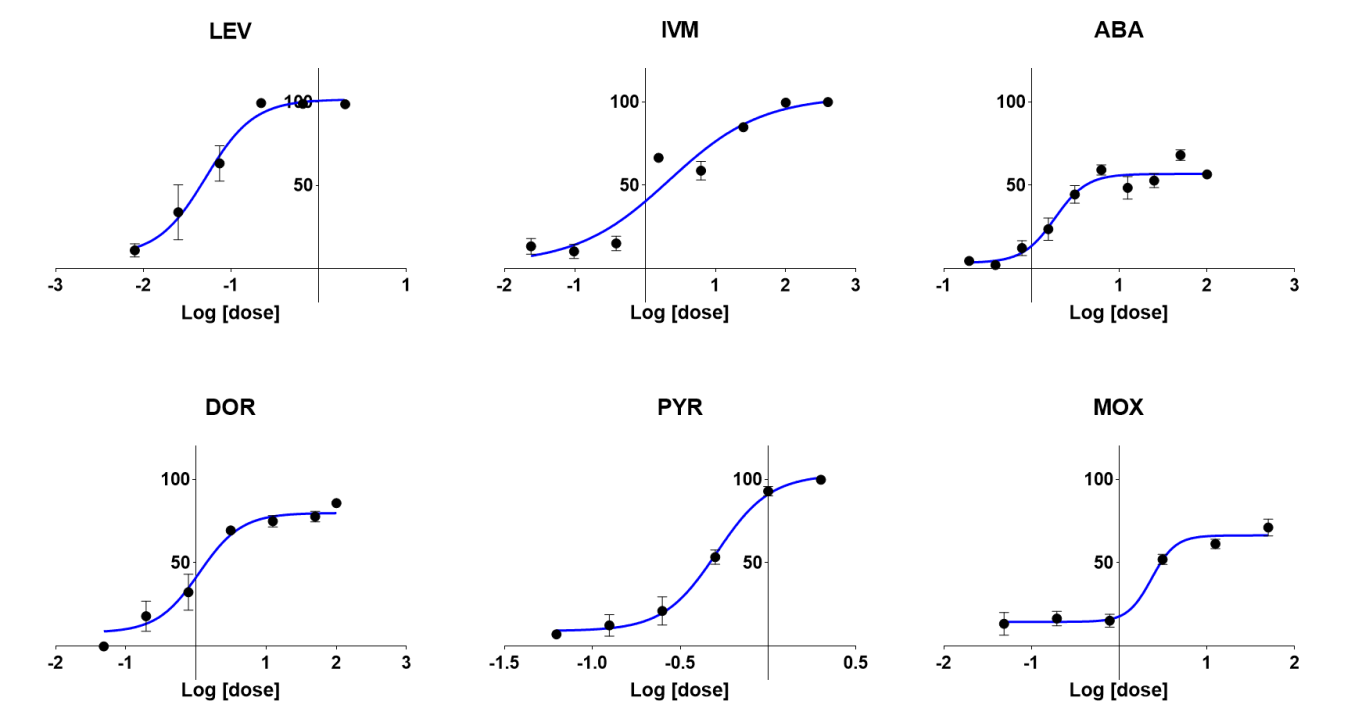


Fig A. Dose-response curves for commercial drugs derived from the Larval Migration Inhibition Test (LMIT). Y-axis is the efficacy percentage (%). The standard error is shown by the error bars.


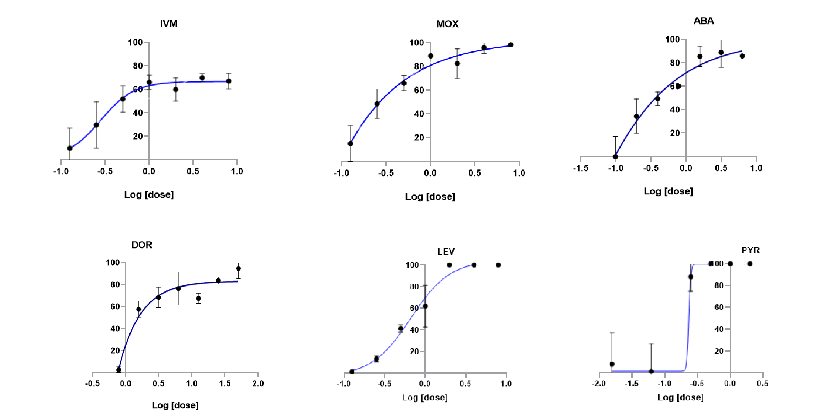


Fig B. Dose-response curves for commercial drugs derived from the Larval Mortality Test (LMT). Y-axis is the efficacy percentage (%). The standard error is shown by the error bars.


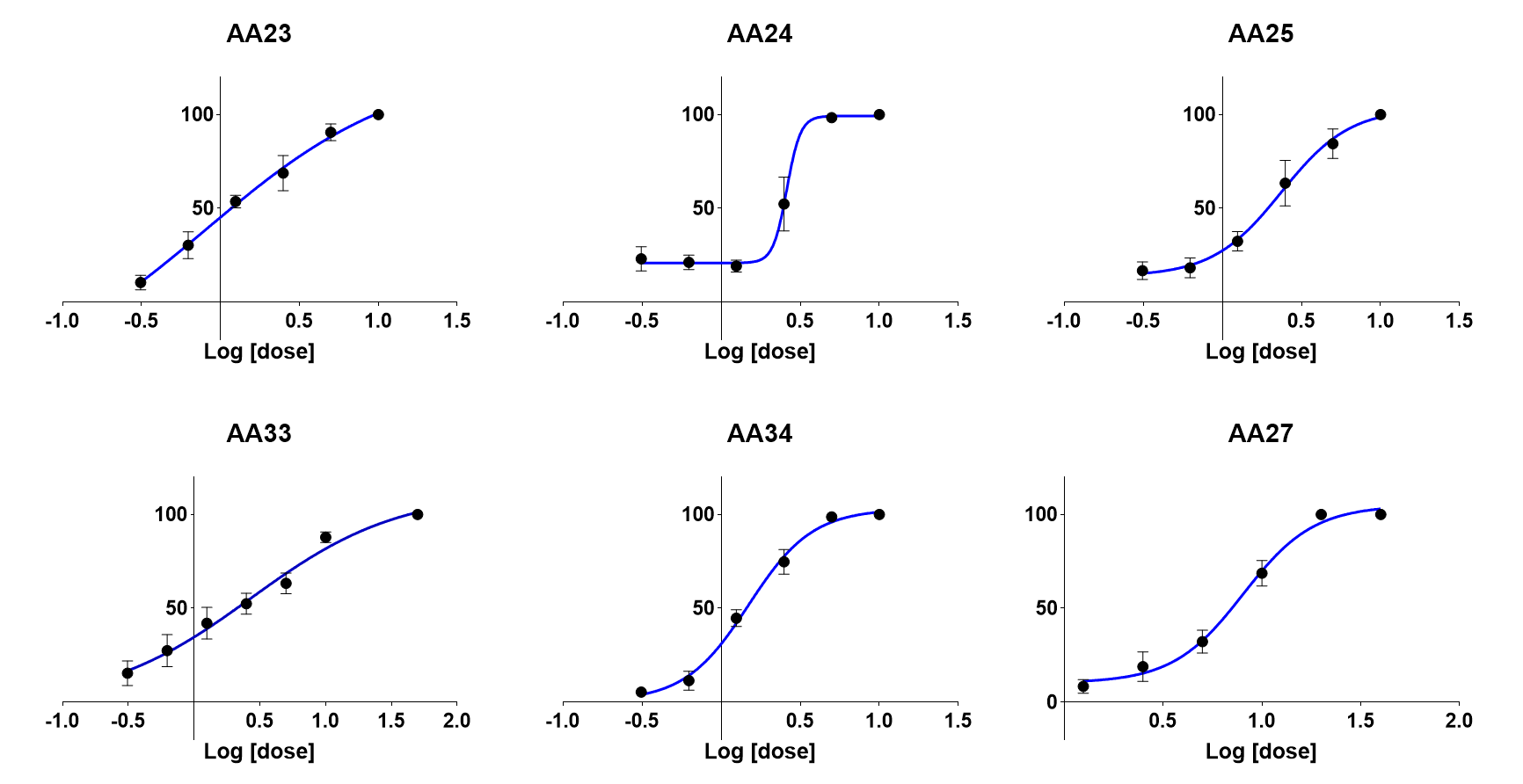


Fig C. Dose-response curves for diamine (AA) derivatives derived from the Larval Migration Inhibition Test (LMIT). Y-axis is the efficacy percentage (%). The standard error is shown by the error bars.


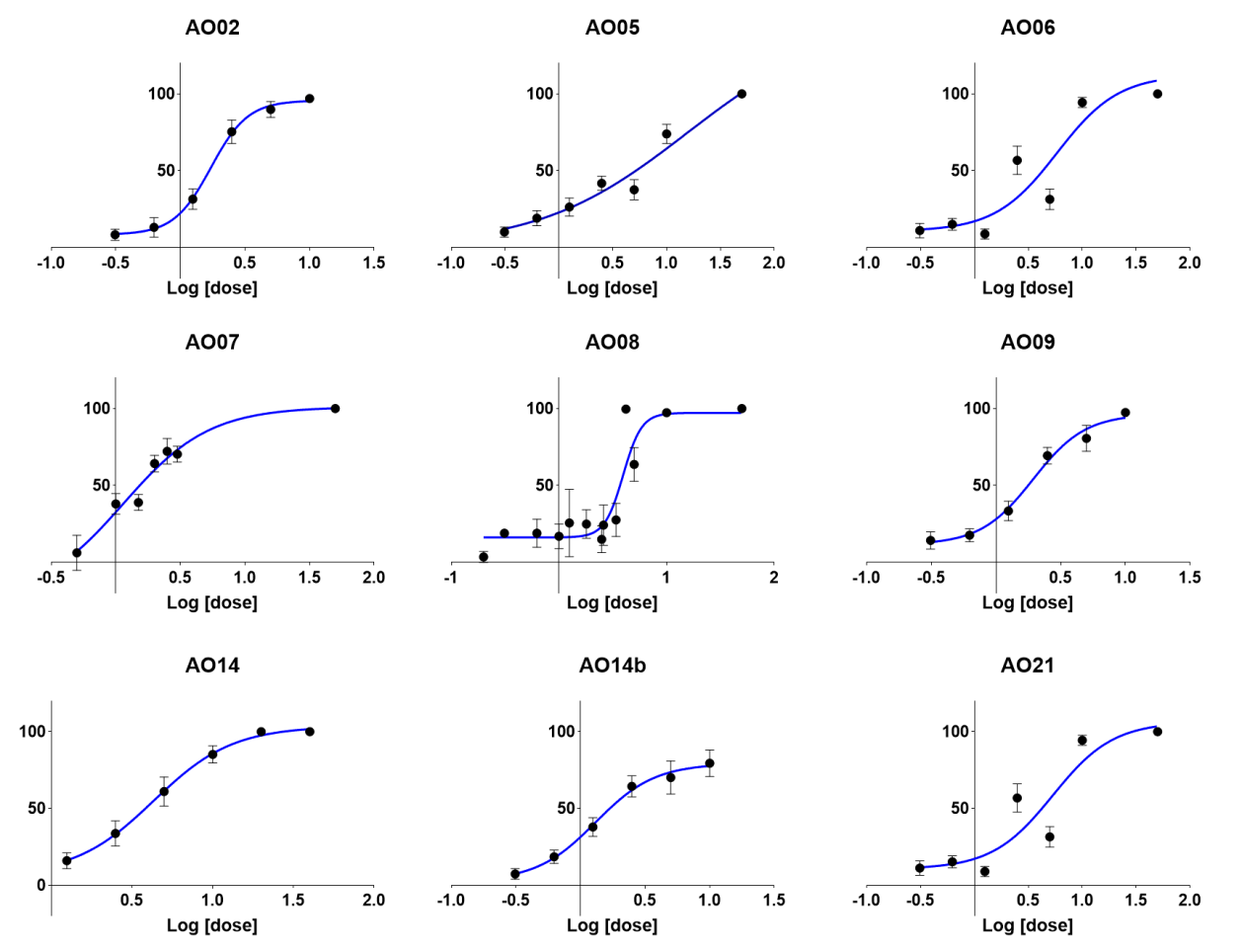


Fig D. Dose-response curves for aminoalcohol (AO) derivatives derived from the Larval Migration Inhibition Test (LMIT). Y-axis is the efficacy percentage (%). The standard error is shown by the error bars.
